# Factors Associated with Visual Acuity in Advanced Glaucoma

**DOI:** 10.3390/jcm12093076

**Published:** 2023-04-24

**Authors:** Hyun Jee Kim, Mi Sun Sung, Sang Woo Park

**Affiliations:** Department of Ophthalmology, Research Institute of Medical Sciences, Chonnam National University Medical School and Hospital, Gwangju 61469, Republic of Korea

**Keywords:** visual acuity, advanced glaucoma, subfoveal choroidal thickness, central visual field

## Abstract

This study aimed to comprehensively analyze various parameters in advanced glaucoma patients to identify the factors that can affect best-corrected visual acuity (BCVA) in advanced glaucoma. This cross-sectional retrospective study included 113 patients (mean age, 61.66 ± 13.26 years; males, 67) who had advanced glaucomatous damage (113 eyes; mean BCVA, 0.18 ± 0.38 logMAR; mean deviation of 30-2 visual field [VF], −19.08 ± 6.23 dB). Peripapillary retinal nerve fiber layer (RNFL) and total and segmented macular thickness (RNFL, ganglion cell layer (GCL), and inner plexiform layer (GCL)) were measured using Spectralis optical coherence tomography (OCT). Correlations between BCVA and OCT parameters or 30-2 VF parameters were assessed using Pearson correlation analysis. Multivariate regression analysis was performed to determine the factors associated with BCVA in advanced glaucoma patients. Peripapillary RNFL thickness, subfoveal choroidal thickness, and global macular RNFL, GCL, IPL, and total thickness were found to be significantly correlated with BCVA and central visual function. Multivariate analysis showed a significant correlation between subfoveal choroidal thickness and BCVA. In addition, central VF mean sensitivity, especially inferior hemifield, showed a significant relationship with BCVA. In conclusion, subfoveal choroidal thickness and central VF sensitivity, especially the inferior hemifield area, are factors that affect BCVA in advanced glaucoma.

## 1. Introduction

Glaucoma is the leading cause of visual dysfunction and blindness worldwide. The disease is associated with various genetic mechanisms and alterations in biochemical pathways that affect the visual system [1]. Therefore, the ultimate purpose of glaucoma treatment is to maintain visual function and vision-related QOL through the early detection and prevention of disease progression [2]. Because of the distinct features of the disease, i.e., it is “chronic and irreversible”, it can significantly affect the quality of life (QOL) of glaucoma patients [3,4]. 

Several parameters are known to be associated with vision-related QOL in glaucoma patients [5,6,7,8]. Chun et al. [5] previously showed that visual acuity is the most important factor in severe glaucoma. Similarly, a previous study performed on the Early Manifest Glaucoma Trial (EMGT) cohort demonstrated that visual acuity is significantly correlated with vision-related QOL, independent of visual field (VF) parameters [6]. The central VF remains relatively intact until the severely advanced stage of the disease due to the surplus of retinal ganglion cells in the macular area [9]. However, as the disease progresses, a reduction in best-corrected visual acuity (BCVA) can occur, and this decline can significantly affect the daily life of glaucoma patients. Hence, treatment strategies for preventing BCVA aggravation are clinically important.

A decrease in BCVA is associated with a decrease in VF parameters [10]. However, in clinical practice, some patients experience a rapid decrease in BCVA even when not in the advanced stage, contrary to our expectations [11]. Meanwhile, some patients maintain relatively good visual acuity despite being in the severely advanced stage [9,10]. This suggests that factors other than the extent of VF defects may contribute to BCVA. Takahashi et al. [9] showed that damage to the papillomacular bundle, a specific area of the retina, was strongly associated with BCVA in glaucoma. It has recently been demonstrated that macular vascular density of the deep layer is independently associated with central visual function in glaucoma patients [12,13,14]. However, information regarding the potential factors influencing visual acuity in advanced glaucoma patients is still limited. Therefore, this study aimed to comprehensively analyze various parameters in glaucoma patients to identify the factors that can affect visual acuity in advanced glaucoma.

## 2. Materials and Methods

### 2.1. Participants

We retrospectively reviewed the medical records of 113 patients (113 eyes) who visited the glaucoma clinic and were diagnosed with advanced glaucoma at the Chonnam National University Hospital between February 2019 and May 2022. The Institutional Review Board of the Chonnam National University Hospital approved this retrospective study and waived the need for informed consent due to the use of de-identified patient data. The present study was conducted in accordance with the Declaration of Helsinki.

Each patient received a detailed consultation and underwent a complete ophthalmic examination at the first outpatient visit, which included slit-lamp examination, manifest refraction, BCVA using the logarithm of the minimum angle resolution (logMAR) units, intraocular pressure (IOP) using Goldmann applanation tonometry, optic nerve head (ONH) and peripapillary retinal nerve fiber layer (RNFL) examinations using both color disc photography and red-free RNFL fundus photography, and VF measurements using the Humphrey Field Analyzer (Carl Zeiss Meditec, Dublin, CA, USA) with the Swedish interactive threshold algorithm standard 30-2 program. The axial length and central corneal thickness were measured using optical low-coherence reflectometry (Lenstar; Haag-Streit AG, Koeniz, Switzerland).

The inclusion criteria for the study were age > 18 years; patients with advanced glaucomatous damage with global mean deviation (MD) below −12 dB on the 30-2 VF test according to the simplified modification of the Hodapp–Anderson–Parrish criteria [15]; BCVA of 20/200 or better; and spherical equivalent refractive error within ± 6.0 D and cylinder refraction within ± 5.0. The exclusion criteria were any history of ocular surgery other than uncomplicated cataract or glaucoma surgery; presence of any media opacities that could affect the BCVA or VF results; presence of retinal disease, including fine epiretinal membrane, diabetic retinopathy, and drusen; or presence of neurological disease or intracranial lesion. We strictly excluded eyes with cataracts or posterior capsular opacity after mydriasis to prevent the potential influence of their effects on BCVA.

### 2.2. Visual Field Examination

All patients underwent the 30-2 VF test twice within 2 months of the initial visit. Our protocols for the first VF results were highly variable and inaccurate. The accuracy of the VF results improved across the repetitions. This is called the “learning effect”. To exclude the learning effect, we used a second VF test for analysis. The same test-spot size (Goldmann size III stimulus) and standard perimetric conditions (background luminance, 31.5 apostilbs) were used for all VF examinations. Near-refractive correction was performed as necessary. The VF measurements were considered reliable if the fixation losses were <20% and the false-positive response rates were <15%. False-negative responses were not included in the exclusion criteria because the presence of several false-negative responses was associated more with the patient’s status than reliability [16]. MD, pattern standard deviation (PSD), VF index (VFI), and foveal sensitivity data were extracted from the VF results. In addition, central VF sensitivity, defined as the central 12 points correlating topographically to the macular area, was analyzed. Each visual sensitivity value of the central 12 test points was converted to a linear scale (1/Lambert value) using the following formula, 1/Lambert = (10)^0.1 × dB^, and the converted linear scale values were subsequently averaged to obtain the central mean sensitivity values. The central 12 points were further divided topographically into two hemispheres, superior and inferior, based on the Garway–Heath map. Detailed protocols for the analysis of the VF results are described in our previous study [17].

### 2.3. Spectral-Domain Optical Coherence Tomography

All patients underwent OCT imaging using SD-OCT (Heidelberg Spectralis SD-OCT; Spectralis software version 6.9.4; Heidelberg Engineering GmbH, Heidelberg, Germany) at the initial visit. All OCT scans were performed by an experienced operator (M.Y.H.). The Bruch membrane opening (BMO) area and peripapillary RNFL thickness (along a 3.5 mm diameter-circle scan) were measured using Glaucoma Module Premium Edition software. The global value and six Garway–Heath regional peripapillary RNFL thickness values relative to the FoBMO axis (the line connecting the center of the fovea and the BMO center) were calculated (nasal-superior, 85–125 degrees; nasal, 125–235 degrees; nasal-inferior, 235–275 degrees; temporal-inferior, 275–315 degrees; temporal, 315–45 degrees; and temporal-superior, 45–85 degrees).

Macular scans were performed using the Posterior Pole algorithm and 30-25-degree volume scans centered on the fovea were acquired. The segmentation of individual retinal layers was performed using the Glaucoma Module Premium Edition software. In addition to the total retinal thickness, the macular RNFL, macular ganglion cell layer (GCL), and macular inner plexiform layer (IPL) were measured. All images from the B-scans were reviewed thoroughly for segmentation errors, and any obvious errors were corrected manually.

### 2.4. Optic Nerve Head Measurements

The Heidelberg Spectralis OCT’s enhanced depth-imaging mode was used for the other ONH measurements. The ONH was scanned by centering a 15 × 10 degree rectangular scan on the ONH. Each OCT volume consisted of 49 serial horizontal B-scans with a length of 4.5 mm in length and 50 images averaged, spaced at approximately 63 μm intervals. Among the B-scans, three sections that passed through the center of the ONH, midsuperior, and midinferior regions were selected for ONH analysis.

The temporal β-parapapillary atrophy margin (β-PPA), BMO, and disc margin were defined using infrared fundus images. The β-PPA width was defined as the distance between the beginning of the retinal pigment epithelium (RPE) (i.e., temporal β-PPA margin) and temporal disc margin on each horizontal B-scan image. Based on the location of the Bruch’s membrane (BM) termination, the β-PPA was further divided into PPA_+BM_ and PPA_−BM_ The PPA_+BM_ width was defined as the distance from the beginning of the RPE to the BM, and the PPA_−BM_ was defined as the distance from the temporal disc margin to the beginning of the BM.

The lamina cribrosa (LC) depth was defined as the vertical distance between the reference line and the anterior LC surface at the center of the ONH from selected horizontal B-scan images. The LC thickness was defined as the perpendicular distance between the anterior and posterior margins of the highly reflective region at the ONH vertical center. In this study, the sclerochoroid junction reference plane was used for the LC depth measurements to overcome the effect of choroidal thickness. The measurement was performed using a built-in caliper tool in the intrinsic OCT viewer, and the average data of three horizontal B-scan images (center, midsuperior, and midinferior) were calculated and used for the analysis. The ONH measurements were performed by two independent examiners (H.J.K. and M.S.S.) blinded to the patients’ information, and the means of the values obtained by the two examiners were used in the final analysis. Details regarding the ONH measurements have been described in previous studies [18,19,20,21].

### 2.5. Choroidal Thickness Measurements

Peripapillary choroidal thickness was measured manually using the embedded software [22]. We delineated the upper and lower segmentation lines of the 360-degree circular peripapillary RNFL scan (3.5 mm), centered on the BMO. The lines were adjusted to align with the inner scleral wall and posterior border of the RPE to define the outer and inner boundaries of the choroid, respectively. The software automatically computed the peripapillary choroidal thickness using the RNFL thickness sector algorithms. Subfoveal choroidal thickness was measured manually from a single EDI scan running through the fovea with the caliper function embedded in the Spectralis instrument. Subfoveal choroidal thickness is defined as the vertical distance from the hyperscattering outer border of the RPE to the inner border of the sclera at the fovea. The average measurements from the two independent examiners (H.J.K. and M.S.S.) were used in this study.

### 2.6. Statistical Analysis

All statistical analyses were performed using SPSS version 23.0 (SPSS, Chicago, IL, USA). Partial correlation analyses were conducted to determine the relationships between the OCT structural parameters and functional parameters such as BCVA and central visual function while adjusting for age. Relationships between the BCVA and several VF parameters were examined using Pearson correlation analyses. The differences in the strength of the correlations between BCVA and the mean sensitivity of the central six points in the superior and inferior hemifields were compared using Steiger’s test [23]. Univariate and multivariate regression analyses were performed to determine the significant factors affecting BCVA in advanced glaucoma patients. The statistical significance was set at *p* < 0.05.

## 3. Results

A total of 113 eyes of 113 patients with advanced glaucoma were included in this cross-sectional retrospective study. The mean age of the patients was 61.66 ± 13.26 years and 57.29% (67 patients) were men. The mean BCVA was 0.18 ± 0.38 logMAR and the mean IOP was 24.96 ± 10.40 mmHg. The MD and PSD values for the 30-2 VF were −19.52 ± 5.51 dB and 12.07 ± 3.43 dB, respectively. The demographic features of the patients with advanced glaucoma are presented in Table 1.

Table 2 shows the results of the partial correlation analysis between the clinical and structural variables and BCVA or central VF sensitivity after adjusting for age in advanced glaucoma patients. Overall, peripapillary RNFL thickness and global macular RNFL, GCL, IPL, and total thickness showed significant correlations with both BCVA and central VF sensitivity (all *p* < 0.05). Furthermore, subfoveal choroidal thickness and the temporal sector of peripapillary choroidal thickness showed significant correlations with the BCVA and VF sensitivity of the central 12 points (*p* = 0.001 and *p* = 0.006, respectively, for BCVA; *p* = 0.045 and *p* = 0.048, respectively, for central VF sensitivity).

Furthermore, we evaluated the association between the 30-2 VF parameters and BCVA in advanced glaucoma patients (Table 3). As expected, as the severity of functional damage increased, patients had worse BCVA. We found a statistically significant correlation in advanced glaucoma patients between the BCVA and functional parameters such as the MD (r = −0.374, *p* < 0.001), PSD (r = −0.397, *p* < 0.001), VFI (r = −0.424, *p* < 0.001), foveal sensitivity (r = −0.549, *p* < 0.001), and mean sensitivity of the central 12 points (r = −0.493, *p* < 0.001). When the central 12 points were further divided into superior and inferior hemifields, both demonstrated significant relationships with BCVA (r = −0.202, *p* = 0.015 for the superior hemifield, and r = −0.445, *p* < 0.001 for the inferior hemifield). However, there was a difference in the strength of the correlations between them; the mean sensitivity of the central six points of the inferior hemifield tended to have a better relationship with BCVA than that of the superior hemifield (*p* = 0.044).

**Figure 1:**
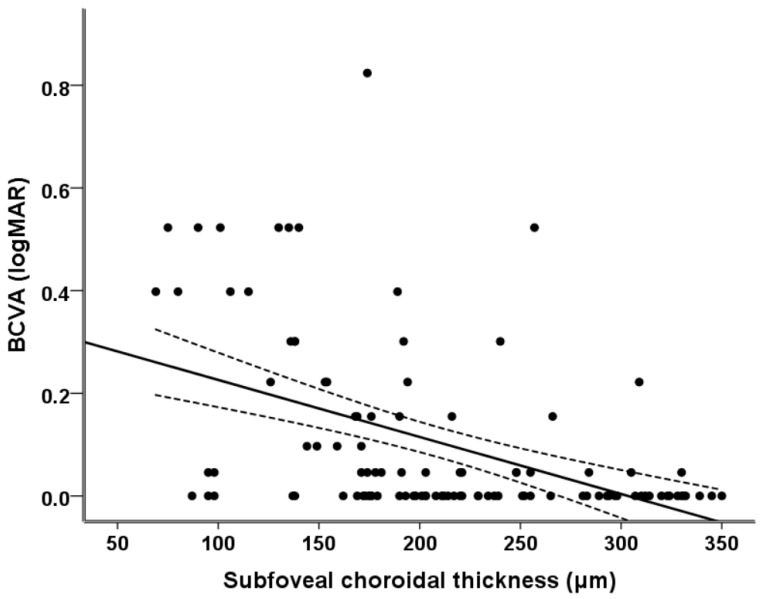
Scatter plots showing the relationship between BCVA and subfoveal choroidal thickness. The dashed lines represent the 95% confidence intervals for the solid trend lines.

To determine the ocular and structural factors that have a close relationship with BCVA in advanced glaucoma patients, univariate and multivariate regression analyses were performed. We eliminated the factors that were not statistically meaningful in the univariate model before analysis. Because the peripapillary RNFL thickness and global macular RNFL, GCL, IPL, and total thickness showed significant multicollinearity, we only used the global macular GCL thickness out of the macular parameters, and the peripapillary RNFL thickness and global macular GCL thickness were used in a different multivariate regression model. The macular GCL thickness was chosen as it showed the strongest correlation with BCVA in the univariate regression analysis. As summarized in Table 4, the PPA_−BM_ width (*p* = 0.001), subfoveal choroidal thickness (*p* = 0.001), peripapillary RNFL thickness (*p* < 0.001), and global macular GCL thickness (*p* < 0.001) showed significant associations with BCVA in the univariate analysis. Through multivariate regression analysis, the subfoveal choroidal thickness (*p* = 0.020 in model 1 and *p* = 0.043 in model 2), peripapillary RNFL thickness (*p* = 0.001), and global macular GCL thickness (*p* < 0.001) were identified as statistically meaningful factors associated with BCVA in advanced glaucoma patients. Figure 1 illustrates the relationship between the subfoveal choroidal thickness and BCVA. It can be seen that as the subfoveal choroidal thickness decreased, the patients’ BCVA became significantly worse (r^2^ = 0.100, r = 0.316, *p* = 0.001).

## 4. Discussion

Central visual function is closely related to patients’ QOL [7]. Thus, maintaining BCVA and central VF is important in glaucoma patients [9]. Either a loss of the central VF or a decrease in the BCVA can significantly affect vision-related QOL [11]. Generally, it is known that central VF is relatively well-preserved until the late stage of glaucoma [24]. However, as the severity of glaucoma increases, a deterioration of central vision inevitably occurs. In clinical situations, the degree of central VF damage and BCVA is highly variable among patients, even in those with similar severity of disease. Specifically, some patients maintain a good BCVA and reserved central VF until the terminal stage of glaucoma, whereas other patients exhibit BCVA deterioration even before advanced glaucomatous damage. In the present study, we focused on the factors affecting BCVA in eyes with advanced glaucomatous damage. Although BCVA is an important factor in determining the visual function and vision-related QOL in glaucoma patients, information regarding BCVA is scarce compared to information regarding VF parameters. Hence, in this study, we focused on BCVA in advanced glaucoma patients and comprehensively investigated the various factors affecting BCVA.

In this study, the peripapillary RNFL thickness and global macular RNFL, GCL, IPL, and total thickness showed significant correlations with BCVA and central visual function. Our findings indicate that as the degree of structural damage increases, a significant decrease in BCVA occurs in patients with advanced glaucoma. These findings are consistent with those of previous studies [9,25,26]. Kim et al. [25] observed significant correlations between the logMAR BCVA and peripapillary RNFL thickness in glaucoma eyes with severe disease status. Among the sectors of the peripapillary RNFL area, Takahashi et al. [9] demonstrated that the temporal sector, which corresponds to the location of the papillomacular bundle, had a strong relationship with BCVA in glaucoma patients. The papillomacular bundle is an axonal fiber bundle emanating from the macula and fovea that carries information from the macula [27,28]. Since visual acuity is dependent on the spatial resolution capacity of the visual system, it is, in turn, influenced by the RGC density at the foveal lesion. Thus, the papillomacular bundle, which is responsible for central visual function, is considered a predictive marker for poor vision [27]. We also found that peripapillary RNFL thickness, especially the temporal sector, and the global macular thickness parameters showed significant associations with BCVA in advanced glaucoma patients.

Of note, the subfoveal choroidal thickness showed a significant correlation with BCVA and central VF sensitivity on 30-2 VF in advanced glaucoma patients. The effect of subfoveal choroidal thickness on BCVA remained statistically significant, even after controlling for the severity of structural damage by multivariate regression analysis. Previously, a report from the Beijing Eye Study, a population-based study of a large Chinese cohort, demonstrated an association between BCVA and subfoveal choroidal thickness [29]. In that study, eyes with thinner subfoveal thickness tended to have lower BCVA, whereas eyes with thicker subfoveal choroidal thickness were associated with better BCVA [29]. In another study, Nishida et al. [30] demonstrated that the only significant predictor of visual acuity in myopic eyes without other retinal pathology was subfoveal choroidal thickness. Although our results cannot be directly compared to those of prior studies because of differences in the study populations, our findings are consistent with those of prior studies.

The outer retinal segment of the fovea, especially the photoreceptor layer, is the most important area for determining BCVA. Moreover, the fovea is mainly nourished by choroidal circulation. Although the role of the choroid in the pathogenesis of glaucoma remains unclear, we suggest that a thin subfoveal choroid may be associated with decreased blood flow in the fovea region, which can lead to photoreceptor dysfunction that subsequently affects the functional status of the overlying outer retina. Recently, associations between macular microvasculature and central visual function or BCVA have been reported by several studies using OCT angiography [12,13,14], which found that macular microvascular density, especially from the deep layer, was an important independent predictor of BCVA [12,13,14]. Additionally, Song et al. [14] demonstrated that the severity of central VF impairment can vary significantly according to the status of deep macular microvasculature, despite similar VF MD and macular structural damage. Since the parafoveal region can support central visual function, impairment of the blood flow at the parafoveal retina can affect central visual function. In terms of highlighting the role of blood flow in the functional status of glaucoma, the findings of Song et al. are consistent with our results. However, the thinning of the choroid may not necessarily indicate the impairment of the blood flow. Meanwhile, Egawa et al. [31] reported a thinning of subfoveal choroid in eyes with retinitis pigmentosa (RP) and found that central visual function was significantly correlated with the ratio of the luminal/total choroidal area representing the inner subfoveal choroidal structure rather than the subfoveal choroidal thickness. Further research is needed to clarify the role of the subfoveal choroid in central visual function in glaucoma patients.

We observed significant relationships between the BCVA and 30-2 VF parameters. Among them, parameters representing the central visual function, such as the VFI, foveal sensitivity, and mean sensitivity of the central 12 points, showed strong correlations with BCVA. Our findings indicate that BCVA can also be used as an important indicator for the progression of glaucoma in patients with advanced disease. When the central 12 points were further divided into superior and inferior hemispheres, the strength of the correlations varied significantly. Interestingly, we found that the mean sensitivity of the central six points in the inferior hemifield tended to have a better relationship with BCVA than that in the superior hemifield in advanced glaucoma patients. Previously, Sawada et al. [8] explored the correlations between vision-specific QOL and clustered VF in glaucoma patients, using the Japanese version of the 25-item National Eye Institute Visual Function Questionnaire (NEI VFQ-25), which comprises 12 subscales related to the status of vision-related activities, social functioning, and emotional well-being. The study found that the VF in the better eye, particularly the lower hemifield, had a stronger correlation with QOL than that in the worse eye. Similarly, Cheng et al. [32] reported that glaucoma patients with superior hemifield VF defects had difficulty in near activities that required close-up vision, whereas patients with inferior hemifield VF defects reported more difficulties with general and peripheral vision and were more likely to report vision-related role difficulties. In our study, BCVA was measured at a distance of 5 m, which is closer to far vision than near vision. Accordingly, the mean sensitivity of the central six points in the inferior hemifield may have a stronger relationship with BCVA. Our findings are clinically significant as they highlight the importance of the inferior central VF for maintaining BCVA in advanced glaucoma patients.

This study has several limitations. First, the sample size was relatively small. Second, some bias may exist because of the study’s retrospective nature. Third, only Korean patients were included in this study and our findings may not be applicable to other ethnicities. Thus, studies including larger and more diverse populations are needed. Fourth, because this study was conducted on patients from tertiary glaucoma clinics, our findings may not reflect the experience of glaucoma patients treated in other clinical settings. Fifth, although we excluded patients with cataracts or retinol lesions that could affect BCVA, several confounding factors may have affected our findings. However, we applied strict inclusion and exclusion criteria to minimize the impact of these factors. Finally, we did not consider systemic conditions, such as blood pressure and diabetes mellitus, or the use of systemic drugs that may affect the visual function of glaucoma patients. Future studies should take into account various systemic factors to further clarify the factors affecting BCVA in glaucoma patients.

In conclusion, the severity of the structural glaucomatous damage, such as peripapillary RNFL thickness and macular RNFL, GCL, IPL, and total thickness, was associated with BCVA in patients with advanced glaucomatous damage. Moreover, the subfoveal choroidal thickness and central VF sensitivity, especially in the inferior hemifield area, are factors that affect BCVA. Clinicians should carefully consider these findings when treating advanced glaucoma patients.

## Figures and Tables

**Table 1:** Demographic data of patients with advanced glaucoma.

Variable	N = 113
Age (years)	61.66 ± 13.26
Sex (male/female)	67/46
Laterality (right/left)	62/51
BCVA (logMAR)	0.18 ± 0.38
Axial length (mm)	24.49 ± 1.47
Central corneal thickness (μm)	529.28 ± 50.43
Baseline IOP (mmHg)	24.96 ± 10.40
OCT parameters	
BMO area (mm^2^)	2.40 ± 0.54
LC thickness (μm)	201.69 ± 36.46
LC depth (μm)	516.93 ± 150.25
PPA_+BM_ width (μm)	251.91 ± 180.14
PPA_−BM_ width (μm)	112.22 ± 192.54
Subfoveal choroidal thickness (μm)	207.69 ± 71.70
Global peripapillary RNFL thickness (μm)	52.16 ± 12.43
Global peripapillary choroidal thickness (μm)	114.18 ± 45.61
Global macular RNFL thickness (μm)	19.81 ± 2.67
Global macular GCL thickness (μm)	30.83 ± 7.32
Global macular IPL thickness (μm)	29.27 ± 4.52
Global macular thickness (μm)	306.99 ± 18.40
30-2 VF parameters	
MD (dB)	−19.52 ± 5.51
PSD (dB)	12.07 ± 3.43
VFI (%)	40.79 ± 19.91
Foveal sensitivity (dB)	27.24 ± 9.77
Mean sensitivity of the central 12 points (dB)	−19.08 ± 6.23

BCVA = best-corrected visual acuity; logMAR = logarithm of the minimum angle of resolution; IOP = intraocular pressure; OCT = optical coherence tomography; BMO = Bruch’s membrane opening; LC = lamina cribrosa; PPA_+BM_ = β-parapapillary atrophy with Bruch’s membrane; PPA_−BM_ = β-parapapillary atrophy without Bruch’s membrane; RNFL = retinal nerve fiber layer; GCL = ganglion cell layer; IPL = inner plexiform layer; VF = visual field; MD = mean deviation; PSD = pattern standard deviation; VFI = visual field index.

**Table 2:** Correlations between clinical and structural variables and best-corrected visual acuity or central visual function in advanced glaucoma patients.

Variable	BCVA		Central VF Sensitivity on 30-2 VF	
	r	*p*-Value *	r	*p*-Value *
Axial length	0.130	0.211	0.158	0.126
Central corneal thickness	0.002	0.987	0.154	0.131
Baseline IOP	0.126	0.184	−0.065	0.495
BMO area	0.032	0.735	0.021	0.826
LC thickness	0.015	0.874	−0.018	0.852
LC depth	−0.166	0.079	0.062	0.511
PPA_+BM_ width	0.003	0.976	−0.072	0.452
PPA_−BM_ width	0.234	**0.013**	0.078	0.414
Subfoveal choroidal thickness	−0.316	**0.001**	0.202	**0.045**
Peripapillary RNFL thickness				
Global	−0.345	**<0.001**	0.387	**<0.001**
Temporal-superior	−0.177	0.060	0.363	**<0.001**
Temporal	−0.393	**<0.001**	0.424	**<0.001**
Temporal-inferior	−0.064	0.499	0.042	0.660
Nasal-inferior	−0.176	0.061	0.086	0.366
Nasal	−0.235	**0.012**	0.234	**0.013**
Nasal-superior	−0.227	**0.015**	0.279	**0.003**
Peripapillary choroidal thickness				
Global	−0.248	**0.008**	0.125	0.189
Temporal-superior	−0.214	**0.023**	0.075	0.430
Temporal	−0.258	**0.006**	0.194	**0.048**
Temporal-inferior	−0.257	**0.006**	0.122	0.199
Nasal-inferior	−0.223	**0.018**	0.108	0.256
Nasal	−0.228	**0.015**	0.096	0.311
Nasal-superior	−0.186	**0.049**	0.158	0.095
Global macular RNFL thickness	−0.376	**<0.001**	0.264	**0.006**
Global macular GCL thickness	−0.473	**<0.001**	0.478	**<0.001**
Global macular IPL thickness	−0.475	**<0.001**	0.413	**<0.001**
Global macular thickness	−0.400	**<0.001**	0.215	**0.026**

BCVA = best-corrected visual acuity; IOP = intraocular pressure; BMO = Bruch’s membrane opening; LC = lamina cribrosa; PPA_+BM_ = β-parapapillary atrophy with Bruch’s membrane; PPA_−BM_ = β-parapapillary atrophy without Bruch’s membrane; RNFL = retinal nerve fiber layer; GCL = ganglion cell layer; IPL = inner plexiform layer; VF = visual field; MD = mean deviation; PSD = pattern standard deviation; VFI = visual field index. * *p* values from partial correlation analysis adjusted by age. Factors with statistical significance are shown in bold.

**Table 3:** Association between BCVA and 30-2 VF parameters in advanced glaucoma patients.

Variable	BCVA	
	r	*p*-Value *
MD	−0.374	**<0.001**
PSD	−0.397	**<0.001**
VFI	−0.424	**<0.001**
Foveal sensitivity	−0.549	**<0.001**
Mean sensitivity of central 12 points	−0.493	**<0.001**
Mean sensitivity of central 6 points in superior hemifield	−0.202	**0.015**
Mean sensitivity of central 6 points in inferior hemifield	−0.445	**<0.001**

BCVA = best-corrected visual acuity; VF = visual field; MD = mean deviation; PSD = pattern standard deviation; VFI = visual field index. * *p* values from Pearson correlation analysis. Factors with statistical significance are shown in bold.

**Table 4:** Multivariate analysis of the associations between BCVA and ocular and structural parameters.

Variable	Univariate			Multivariate (Model 1)			Multivariate (Model 2)		
	Coefficient	95% CI	*p*-Value *	Coefficient	95% CI	*p*-Value *	Coefficient	95% CI	*p*-Value *
PPA_−BM_ width	0.001	0.000–0.001	**0.001**	0.00038	0.000–0.001	0.056	0.00025	0.000–0.001	0.120
Subfoveal choroidal thickness	−0.002	−0.003–−0.001	**0.001**	−0.001	−0.002–0.000	**0.020**	−0.001	−0.002–0.000	**0.043**
Peripapillary RNFL thickness	−0.010	−0.016–−0.005	**<0001**	−0.009	−0.014–−0.004	**0.001**			
Global macular GCL thickness	−0.023	−0.031–−0.015	**<0.001**				−0.020	−0.029–−0.012	**<0.001**

BCVA = best-corrected visual acuity; CI = confidence interval; PPA_−BM_ = β-parapapillary atrophy without Bruch’s membrane; RNFL = retinal nerve fiber layer; GCL = ganglion cell layer. * *p* values from multivariate regression analysis. Factors with statistical significance are shown in bold.

## Data Availability

The datasets used and/or analyzed during the current study are available from the corresponding author on reasonable request.

## References

[1] Surgucheva I., Park B.-C., Yue B.Y.J.T., Tomarev S., Surguchov A. (2005). Interaction of myocilin with gamma-synuclein affects its secretion and aggregation. Cell Mol. Neurobiol..

[2] Quigley H.A. (2011). Glaucoma. Lancet Lond. Engl..

[3] Pascolini D., Mariotti S.P. (2012). Global estimates of visual impairment: 2010. Br. J. Ophthalmol..

[4] Guedes R.A.P., Guedes V.M.P., Freitas S.M., Chaoubah A. (2013). Quality of Life of Medically Versus Surgically Treated Glaucoma Patients. Eur. J. Gastroenterol. Hepatol..

[5] Chun Y.S., Sung K.R., Park C.K., Kim H.K., Yoo C., Kim Y.Y., Park K.H., Kim C.Y., Choi K., Lee K.W. (2019). Factors influencing vision-related quality of life according to glaucoma severity. Acta Ophthalmol..

[6] Peters D., Heijl A., Brenner L., Bengtsson B. (2015). Visual impairment and vision-related quality of life in the Early Manifest Glaucoma Trial after 20 years of follow-up. Acta Ophthalmol..

[7] Murata H., Hirasawa H., Aoyama Y., Sugisaki K., Araie M., Mayama C., Aihara M., Asaoka R. (2013). Identifying areas of the visual field important for quality of life in patients with glaucoma. PLoS ONE.

[8] Sawada H., Yoshino T., Fukuchi T., Abe H. (2014). Assessment of the Vision-specific Quality of Life Using Clustered Visual Field in Glaucoma Patients. Eur. J. Gastroenterol. Hepatol..

[9] Takahashi N., Omodaka K., Kikawa T., Akiba M., Nakazawa T. (2021). Association between Topographic Features of the Retinal Nerve Fiber Bundle and Good Visual Acuity in Patients with Glaucoma. Curr. Eye Res..

[10] Asaoka R. (2013). The relationship between visual acuity and central visual field sensitivity in advanced glaucoma. Br. J. Ophthalmol..

[11] Sugisaki K., Inoue T., Yoshikawa K., Kanamori A., Yamazaki Y., Ishikawa S., Uchida K., Iwase A., Araie M. (2022). Factors Threatening Central Visual Function of Patients with Advanced Glaucoma: A Prospective Longitudinal Observational Study. Ophthalmology.

[12] Jeon S.J., Park H.-Y.L., Park C.K. (2018). Effect of Macular Vascular Density on Central Visual Function and Macular Structure in Glaucoma Patients. Sci. Rep..

[13] Hsia Y., Wang T.-H., Huang J.-Y., Su C.-C. (2021). Relationship Between Macular Microvasculature and Visual Acuity in Advanced and Severe Glaucoma. Am. J. Ophthalmol..

[14] Song W.K., Kim K.E., Yoon J.Y., Lee A., Kook M.S. (2022). Association of macular structure, function, and vessel density with foveal threshold in advanced glaucoma. Sci. Rep..

[15] Hodapp E., Parrish R., Anderson D.R. (1993). Clinical Decisions in Glaucoma.

[16] Bengtsson B., Heijl A. (2000). False-negative responses in glaucoma perimetry: Indicators of patient performance or test reliability?. Invest. Ophthalmol. Vis. Sci..

[17] Sung M.S.M., Ji Y.S.M., Heo H.M., Park S.W.M. (2022). Comparison of the Structure-Function Relationship between Advanced Primary Open Angle Glaucoma and Normal Tension Glaucoma. Eur. J. Gastroenterol. Hepatol..

[18] Sung M.S., Heo H., Piao H., Guo Y., Park S.W. (2020). Parapapillary atrophy and changes in the optic nerve head and posterior pole in high myopia. Sci. Rep..

[19] Kim Y.W., Lee E.J., Kim T.-W., Kim M., Kim H. (2014). Microstructure of β-Zone Parapapillary Atrophy and Rate of Retinal Nerve Fiber Layer Thinning in Primary Open-Angle Glaucoma. Ophthalmology.

[20] Yamada H., Akagi T., Nakanishi H., Ikeda H.O., Kimura Y., Suda K., Hasegawa T., Yoshikawa M., Iida Y., Yoshimura N. (2016). Microstructure of Peripapillary Atrophy and Subsequent Visual Field Progression in Treated Primary Open-Angle Glaucoma. Ophthalmology.

[21] Lee S.H., Lee E.J., Kim T.-W. (2016). Topographic Correlation Between Juxtapapillary Choroidal Thickness and Microstructure of Parapapillary Atrophy. Ophthalmology.

[22] Sung M.S., Jin H.N., Park S.W. (2021). Clinical Features of Advanced Glaucoma With Optic Nerve Head Prelaminar Schisis. Am. J. Ophthalmol..

[23] Steiger J.H. (1980). Tests for comparing elements of a correlation matrix. Psychol. Bull..

[24] Weber J., Schultze T., Ulrich H. (1989). The visual field in advanced glaucoma. Int. Ophthalmol..

[25] Kim J.H., Lee H.S., Kim N.R., Seong G.J., Kim C.Y. (2014). Relationship Between Visual Acuity and Retinal Structures Measured by Spectral Domain Optical Coherence Tomography in Patients with Open-Angle Glaucoma. Investig. Opthalmol. Vis. Sci..

[26] Suzuki Y., Kiyosawa M. (2021). Visual Acuity in Glaucomatous Eyes Correlates Better with Visual Field Parameters than with OCT Parameters. Curr. Eye Res..

[27] Baek S.U., Lee W.J., Park K.H., Choi H.J. (2021). Health screening program revealed risk factors associated with development and progression of papillomacular bundle defect. EPMA J..

[28] Leung C.K.S., Guo P.Y., Lam A.K.N. (2022). Retinal Nerve Fiber Layer Optical Texture Analysis: Involvement of the Papillomacular Bundle and Papillofoveal Bundle in Early Glaucoma. Ophthalmology.

[29] Shao L., Xu L., Bin Wei W., Chen C.X., Du K.F., Li X.P., Yang M., Wang Y.X., You Q.S., Jonas J.B. (2014). Visual Acuity and Subfoveal Choroidal Thickness: The Beijing Eye Study. Am. J. Ophthalmol..

[30] Nishida Y., Fujiwara T., Imamura Y., Lima L.H., Kurosaka D., Spaide R.F. (2012). Choroidal thickness and visual acuity in highly myopic eyes. Retina.

[31] Egawa M., Mitamura Y., Niki M., Sano H., Miura G., Chiba A., Yamamoto S., Sonoda S., Sakamoto T. (2019). Correlations between choroidal structures and visual functions in eyes with retinitis pigmentosa. Retina.

[32] Cheng H.-C., Guo C.-Y., Chen M.-J., Ko Y.-C., Huang N., Liu C.J.-L. (2015). Patient-Reported Vision-Related Quality of Life Differences Between Superior and Inferior Hemifield Visual Field Defects in Primary Open-Angle Glaucoma. JAMA Ophthalmol..

